# Long Term Liver Engraftment of Functional Hepatocytes Obtained from Germline Cell-Derived Pluripotent Stem Cells

**DOI:** 10.1371/journal.pone.0136762

**Published:** 2015-08-31

**Authors:** Sharmila Fagoonee, Elvira Smeralda Famulari, Lorenzo Silengo, Emanuela Tolosano, Fiorella Altruda

**Affiliations:** 1 Institute for Biostructures and Bioimages (CNR), Molecular Biotechnology Center, Department of Molecular Biotechnology and Health Sciences, University of Turin, Turin, Italy; 2 Molecular Biotechnology Center, Department of Molecular Biotechnology and Health Sciences, University of Turin, Turin, Italy; Montana State University, UNITED STATES

## Abstract

One of the major hurdles in liver gene and cell therapy is availability of *ex vivo*-expanded hepatocytes. Pluripotent stem cells are an attractive alternative. Here, we show that hepatocyte precursors can be isolated from male germline cell-derived pluripotent stem cells (GPSCs) using the hepatoblast marker, Liv2, and induced to differentiate into hepatocytes *in vitro*. These cells expressed hepatic-specific genes and were functional as demonstrated by their ability to secrete albumin and produce urea. When transplanted in the liver parenchyma of partially hepatectomised mice, Liv2-sorted cells showed regional and heterogeneous engraftment in the injected lobe. Moreover, approximately 50% of Y chromosome-positive, GPSC-derived cells were found in the female livers, in the region of engraftment, even one month after cell injection. This is the first study showing that Liv2-sorted GPSCs-derived hepatocytes can undergo long lasting engraftment in the mouse liver. Thus, GPSCs might offer promise for regenerative medicine.

## Introduction

Liver transplantation is presently the only proven treatment for many liver diseases but is not exempt from complications, thus increasing the need for a new approach using exogenous cell transplantation to restore liver functions[1]. Experiments on animals have been performed to establish models and methodologies for liver repopulation using cells other than adult hepatocytes, including hepatic cell lines and hematopoietic, cord blood, mesenchymal stem cells, hepatic stem cells [2]. However, when these cells are cryopreserved, a loss in viability is observed [3, 4]. Pluripotent stem cells like embryonic stem (ES) cells and induced pluripotent stem (iPS) cells have been employed in hepatocyte differentiation *in vitro*[5, 6]. Both mouse and human cells have been used for this purpose [7, 8]. However, several problems are associated with the use of these cells. ES cells are subject to ethical debates, and because of their allogenic nature, transplantation of ES cell-derived hepatocytes requires immunosuppression in the recipient. On the other hand, iPS cells still face hurdles like finding viral-free modes of producing them or their spontaneous tumorigenicity which limit the clinical application. There is thus the need to find alternative sources of cells for liver repair.

In the last decade, pluripotent stem cells derived from spermatogonial stem cells (SSCs) have been much studied as a potential alternative to these above-mentioned cells[9]. Mouse SSCs can be isolated and cultured on mouse embryonic fibroblasts. In long term cultures, these SSCs convert spontaneously to ES-like cells, termed germline cell-derived pluripotent stem cells or GPSCs, and express pluripotency markers like Oct4, Nanog and Sox2, show positivity for stage-specific antigen 1 (SSEA-1) and for alkaline phosphatase, and generate teratomas *in vivo*[10, 11]. Importantly, GPSCs are derived from post-natal or adult mice, and no particular manipulation is required to convert the SSCs to GPSCs *in vitro*. GPSCs show high plasticity and several authors have shown that these cells can be induced to differentiate into functional cardiomyocytes, neurons, hematopoietic cells[12–14]. Recently, we demonstrated that functional renal tubular cells can be obtained from GPSCs and can protect mice against kidney ischemia reperfusion injury[15].

We have previously reported that functional hepatocyte-like cells can be generated from GPSCs *in vitro* [16]. Moreover, a large scale gene expression profiling, performed on GPSCs induced to differentiate into hepatocytes at different time points compared to primary hepatocytes, revealed that the GPSC-derived hepatocytes were closer to fetal hepatocytes than post-natal ones[16]. In view of the potential clinical application of GPSCs, it is imperative to assess whether these cells can home to and engraft in mouse livers *in vivo*. In the present paper, we used anti-Liv2 antibodies, first reported by Watanabe *et al*., to sort for hepatoblasts from GPSC-derived embryoid bodies (EBs) [17]. This antibody was produced upon immunisation of mice with murine liver lysates and recognizes specifically a surface antigen, Liv2 (no gene encoding this antigen has been described hitherto), on mouse hepatoblasts between embryonic days (E) 9.5 to 12.5. Hepatoblasts are bipotential cells that develop into either hepatocytes or cholangiocytes as embryonic development proceeds. We induced the GPSC-derived Liv2-positive hepatoblasts to differentiate into mature hepatocytes *in vitro* and show, for the first time, that these cells are able to engraft in mouse liver after partial hepatectomy.

## Materials and Methods

### Culture of GPSCs and hepatocyte differentiation

**Fig 1:**
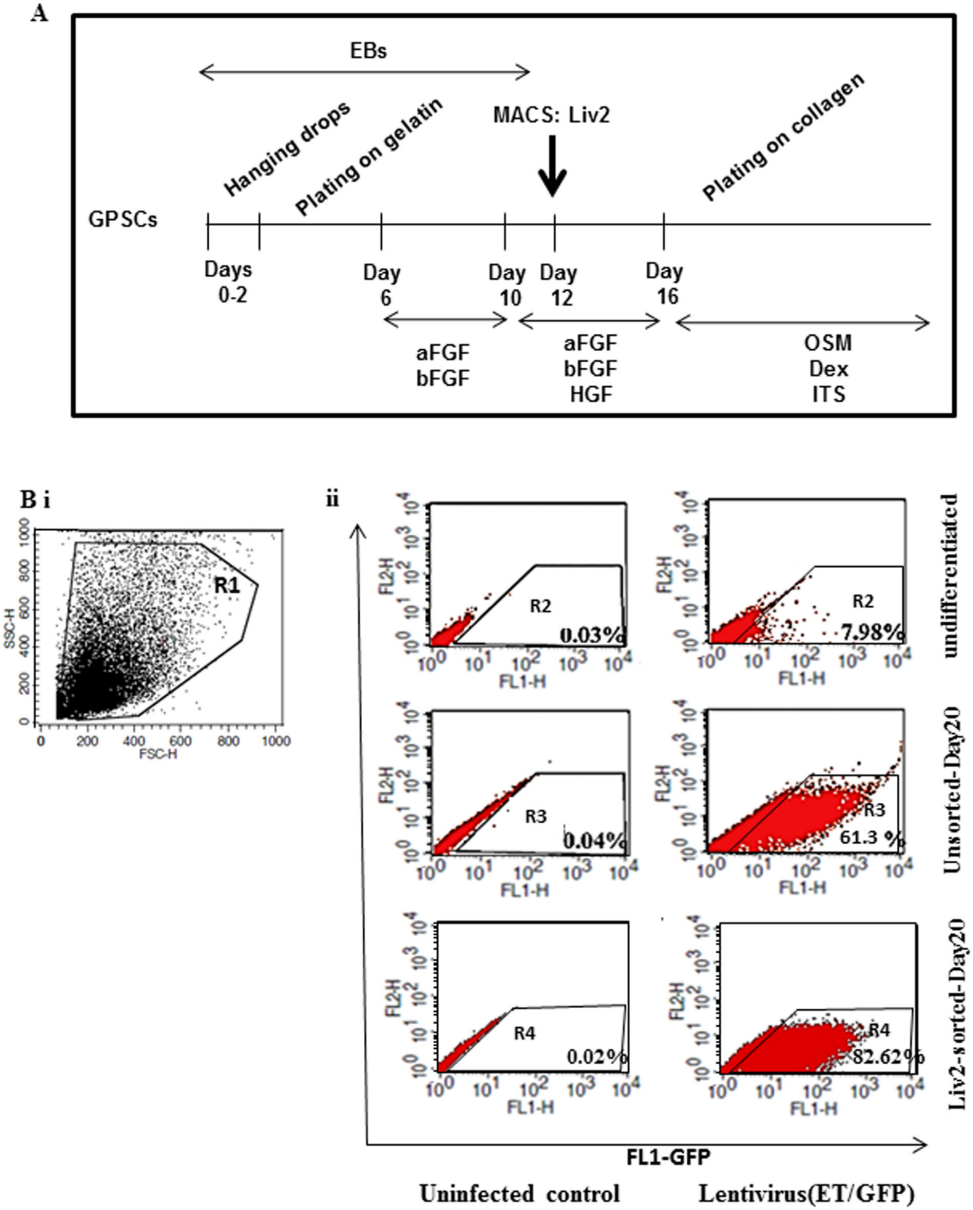
Liv2-positive cell sorting from GPSC-derived EBs. **A.** Procedure for MACS sorting of Liv2-positive cells and hepatocyte differentiation *in vitro*. Liv2-positive cells were sorted from GPSC-derived 13 days old EBs and further differentiated into hepatocytes *in vitro*. **B.** A forward-and-side-scatter plot with gating on live cells (R1) is shown (i). Unsorted GPSCs under basal conditions (undifferentiated), and after 20 days of differentiation (Unsorted) as well as Liv2-sorted GPSC derived hepatocytes (Liv2-sorted) at day 20 of induced differentiation (ii) were infected with pCCL-ET.GFP virus which expresses GFP specifically in hepatocytes and analysed by flow cytometry. GFP fluorescence (FL1) is shown for negative controls (uninfected control) and for the infected samples (Lentivirus (ET/GFP)). Regions 2, 3, and 4 (R2, R3 and R4) were designed to measure GFP fluorescence in infected cells *versus* non-infected controls.

GPSCs (129Sv/C57B (H2^b^)), derived from mouse SSCs, were cultured and induced to differentiate into hepatocytes in IMDM complete media containing IMDM-Glutamax (Invitrogen), 9% FCS, 300 μmol/L mercaptoethanol, 100 U/ml penicillin, 100 μg/ml Streptomycin, 1mM sodium pyruvate, and 1x non-essential amino acids (NEAA) (Invitrogen). Feeder-free GPSCs were cultured in hanging drops (300 cells/drop) in the absence of LIF, and at Day 2, embryoid bodies (EBs) were plated on gelatin for further differentiation. The following factors were added: 20ng/ml acidic fibroblast growth factor (FGF) and 10ng/ml basic FGF from Day 6; 10ng/ml rat recombinant hepatocyte growth factor (HGF, Peprotech) from Day 10; 10ng/ml recombinant mouse oncostatin M (R&D systems), 10^−7^ M dexamethasone and 1x ITS solution (Sigma) from Day 16 (Fig 1A)[16]. At Day 13, EBs were trypsinised for cell sorting as described below.

### Immunohistochemistry for Liv2 and MACS cell sorting

For immunohistochemistry (IHC), EBs were grown in chamber slides. At 11, 13 and 15 differentiation days, EBs were stained with anti-mouse Liv2 antibody (MBL) and revealed with biotinylated anti-rat antibody and the ABC complex (DAKO). Liv2-positive cells were sorted at Day 13 from EBs using magnetic activated cell sorting (MACS, Miltenyi Biotec). EBs were trypsinised and incubated with the primary antibody for 30 minutes followed by incubation with an anti-rat biotinylated secondary antibody for 20 minutes and streptavidin beads for 15 minutes. After elution, Liv2-positive cells were plated and allowed to further differentiate in the hepatocyte differentiation medium as previously described[16].

### HFigepatic gene expression analysis

RNA was extracted using the Purelink RNA kit (Ambion). Following treatment with RQ1 DNAse (Promega), 1μg of RNA was reversed transcribed using the high capacity cDNA reverse transcription kit (Applied Biosystems) and random primers. Primers used for RT-PCR are as previously described[16] while primers for quantitative qRT-PCR were designed using the Universal ProbeLibrary Assay Design Center (Roche) and spanned exon-exon junctions (Table 1). Postnatal hepatocytes were used as positive control and gene expression was normalized to that of 18S.

**Table 1:** Primers used for qRT-PCR in this study were designed using the Roche UPL library.

Gene Name	Left primer	Right primer
**Dlk1**	cgggaaattctgcgaaatag	tgtgcaggagcattcgtact
**CK18**	agatgacaccaacatcacaagg	tccagaccttggacttcctc
**Oct4**	gttggagaaggtggaaccaa	ctccttctgcagggctttc
**Cyp7a1**	tcaagcaaacaccattcctg	ggctgctttcattgcttca
**TNFRSF1A**	tgcaagacatgtcggaaaga	caggtagcgttggaactggt
**IL6RA**	atcctctggaaccccacac	gaactttcgtactgatcctcgtg
**G6Pc**	tctgtcccggatctaccttg	gaaagtttcagccacagcaa

### Lentiviral infection of EBs and flow cytometric analysis

The lentivirus pCCL.ET.GFP.sin, which expresses GFP under the control of the enhanced transthyretin promoter, was produced in 293FT cells as previously described[16]. After MACS sorting, Liv2-sorted cells were infected with viral supernatant containing 8μg/ml polybrene and plated on gelatin. Positivity for GFP was assessed by flow cytometry at Day 20. A total of at least 10 000 cells were analyzed on a FACSCalibur using the CellQuest software (Beckton Dickinson). Gates were set on the forward-to-side scatter (FSC versus SSC) linear dot plot, and the GFP fluorescence (FL1) was measured in infected cells versus non-infected controls.

### Immunofluorescence and immunohistochemistry analysis

Liv2-sorted GPSC derived hepatocytes were incubated with rabbit anti-Ck18, -Dlk1, -albumin, -Ki67, -e-cadherin antibodies (Abcam) overnight at 4°C and according to the manufacturer’s instructions. Alexa Fluor 568-conjugated goat anti-rabbit IgG (Invitrogen) was used as secondary antibody. DAPI was used to stain nuclei. The samples were examined with a Zeiss microscope (Apotome software). Mice livers were fixed in formalin and embedded in paraffin. The liver sections were stained with mouse anti-PCNA (proliferating cell nuclear antigen) (Santa Cruz Biotechnology) using the M.O.M. (mouse on mouse) kit and revealed using biotinylated anti-mouse secondary antibody (Vector Laboratories) and the ABC complex (DAKO). Staining of teratoma sections with anti-Liv2 antibodies was performed as described above.

### Albumin secretion

Medium was collected from EBs grown on collagen gel at indicated time points and assessed for albumin secretion with a mouse albumin ELISA quantitation kit (Bethyl). The plate was read at 450 nm for TMB using a microplate reader (Mithras) as previously described[16].

### Urea production

At Days 18 and 21 of differentitation, one EB was plated per well on matrigel and ammonium chloride was added for 24 hours at a final concentration of 5mM in IMDM complete media supplemented with 10ng/ml recombinant mouse oncostatin M (R&D systems), 10^−7^ M dexamethasone and 1x ITS solution (Sigma). Urea production was determined at various time points using a colorimetric assay (Quantichrom) following the manufacturers’ instructions.

### Periodic acid-Shiff (PAS) staining

At Day 21 of differentiation, EBs were stained with PAS reagents according to the manufacturers’ instructions (Bio Optica).

### Hepatectomy and cell transplantation in mice

Two months old 129Sv (H2^b^) female Hfe-null mice (hereditary hemochromatosis model) were used for experiments and maintained on a standard chow diet and kept with free access to food and water. The experiments were approved by the local animal ethical committee (comitato di bioetica d’ateneo, University of Turin, Italy) and all efforts were made to minimize suffering. Mice were treated intraperitoneally with monocrotaline (50mg/kg) twice (at a 2-week interval) to inhibit endogenous hepatocyte proliferation[18]. Two weeks after the last injection, mice were anesthetized using a mixture of zoletil and rompun and 2 liver lobes were excised (equivalent to 50% hepatectomy). During the same surgery, 2X10^5^ Liv2-sorted GPSC-derived hepatocytes were injected in one of the remaining lobes. Mice were left to recover and then sacrificed at 5 days or one month after cell injection by CO_2_ inhalation followed by cervical dislocation. Undifferentiated GPSCs were also injected in partially hepatectomised livers to assess teratoma formation.

### FISH for Y chromosome

FISH for Y chromosome was performed using mouse Y chromosome (catalogue *#* 1189-YMF-02; Cambio) according to the manufacturer’s protocol. We also synthesised a biotinylated probe as previously described with the following modifications[15]. Formalin-fixed and paraffin-embedded liver sections (3.5 μm) were treated with citrate buffer (pH 6) at 80°C for 90 minutes for antigen retrieval. Sections were denatured at 70°C for 5 minutes and then hybridized with the probe at 37°C for 19 hours. The biotinylated dUTPs were revealed with cyanine (Cy)3-conjugated streptavidin (Jackson ImmunoResearch Laboratories, Inc.). To estimate the percentage of Liv2-sorted cells that engrafted in the transplanted lobe at 1 month after partial hepatectomy, we counted the number of Y chromosome-positive nuclei/total nuclei in 10 fields (138x104 μm^2^) in the regenerating areas using the Z-stack option (63x magnification) of the AxioVision software (Apotome attachment, Zeiss microscope). A male mouse liver was used as positive control.

### Statistical analysis

Graphical representation of individual data is shown. Where specified, data are expressed as mean ± SD and statistical differences were determined by a two-tailed Student’s t test (*P<0.05, **P<0.01, ***P<0.001). All experiments were performed independently at least three times.

## Results

### Characterisation of Liv2-positive GPSC-derived hepatocytes

Liv2 expression was analysed in EBs at different time points and was found to be expressed as from Day 11 to Day 15 in the outgrowths (S1 Fig, arrowheads). Liv2-positive hepatoblasts were MACS-sorted from GPSC-derived EBs at Day 13 and plated on gelatin or collagen as illustrated in Fig 1A. Differentiation was allowed to proceed as previously described[16].

### Enrichment of hepatocytes in Liv2-sorted GPSCs

In order to analyse the percentage of hepatocyte-like cells present in the cultures after Liv2 sorting, we infected the cells with a lentivirus expressing GFP under the enhanced transthyretin promoter. Flow cytometry analysis showed that at Day 20 of differentiation, 82.62% of the Liv2-sorted cells were GFP- positive compared to 7.98% of undifferentiated controls or 61.3% of unsorted GPSC-derived hepatocytes (Fig 1B).

### Liv2-sorted GPSC-derived hepatocytes are functional

**Fig 2:**
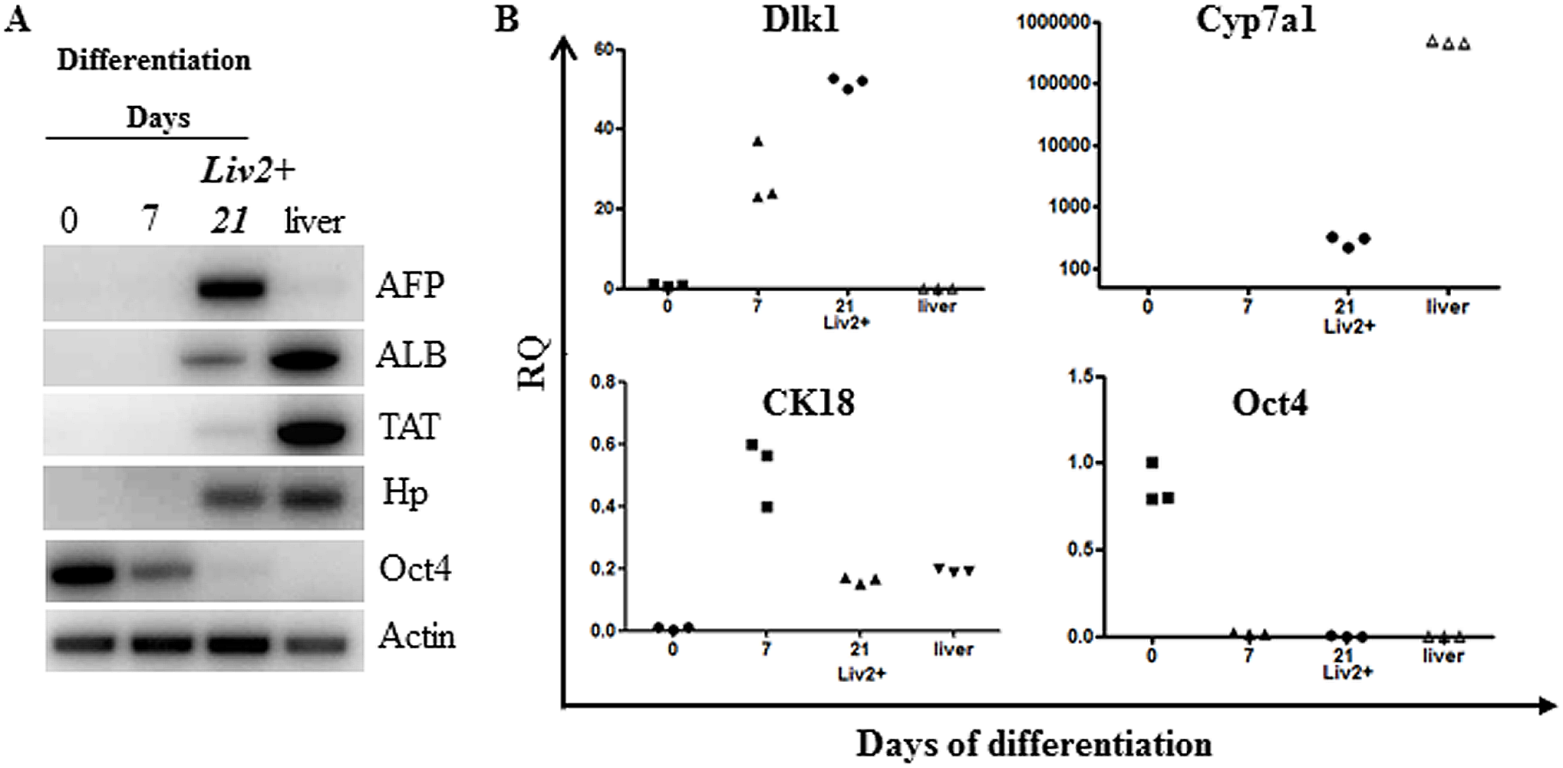
Analysis of hepatocyte-specific gene expression in Liv2-sorted GPSC-derived hepatocytes. **A.** RT-PCR analysis of liver-specific gene expression. RNA samples were extracted from undifferentiated GPSCs cells (0 day), differentiating EBs (7 days), Liv2-sorted cells (21 days) and adult liver. Actin served as an internal standard. Data shown are representative of 3 independent experiments. B. qRT-PCR was also used to assess the expression of Dlk1, Cyp7a1, CK18 and Oct4 in Liv2-sorted GPSCs during hepatocyte differentiation (n = 3). The relative quantity (RQ) with respect to gene expression in undifferentiated GPSCs (differentiation Day 0) is shown and values have been normalized to 18S expression (n = 3). Abbreviations: ALB, albumin; AFP, alpha fetoprotein; TAT, tyrosine amino transferase; Hp, haptoglobin; Dlk1, Delta-like 1 homolog; Cyp7a1, cytochrome P450 7a1; CK18, cytokeratin 18.

Next, we wanted to verify whether Liv-2 sorting did not affect the properties of the previously described GPSC-derived hepatocytes[16]. Liv2-sorted GPSC-derived hepatocytes were tested for their ability to express hepatocyte-specific genes. At Day 21 of differentiation, Liv2-sorted GPSC-derived hepatocytes expressed alpha fetoprotein (AFP), albumin (ALB), tyrosine aminotransferase (TAT), haptoglobin (Hp) compared to the undifferentiated GPSCs or at differentiation Day 7 (Fig 2A). Moreover, these cells expressed delta-like homolog 1 (Dlk1), cytokeratin 18 (CK18), the liver-specific cytochrome P7a1 (Cyp7a1) (Fig 2B). The expression of the pluripotency marker, octamer-binding transcription factor 4 (Oct4), was absent in Liv2-sorted hepatocytes compared to undifferentiated GPSCs. Interestingly, Liv2-sorted GPSCs also express the Hfe (hemochromatosis) gene as from differentiation Day 14 (S2 Fig)

**Fig 3:**
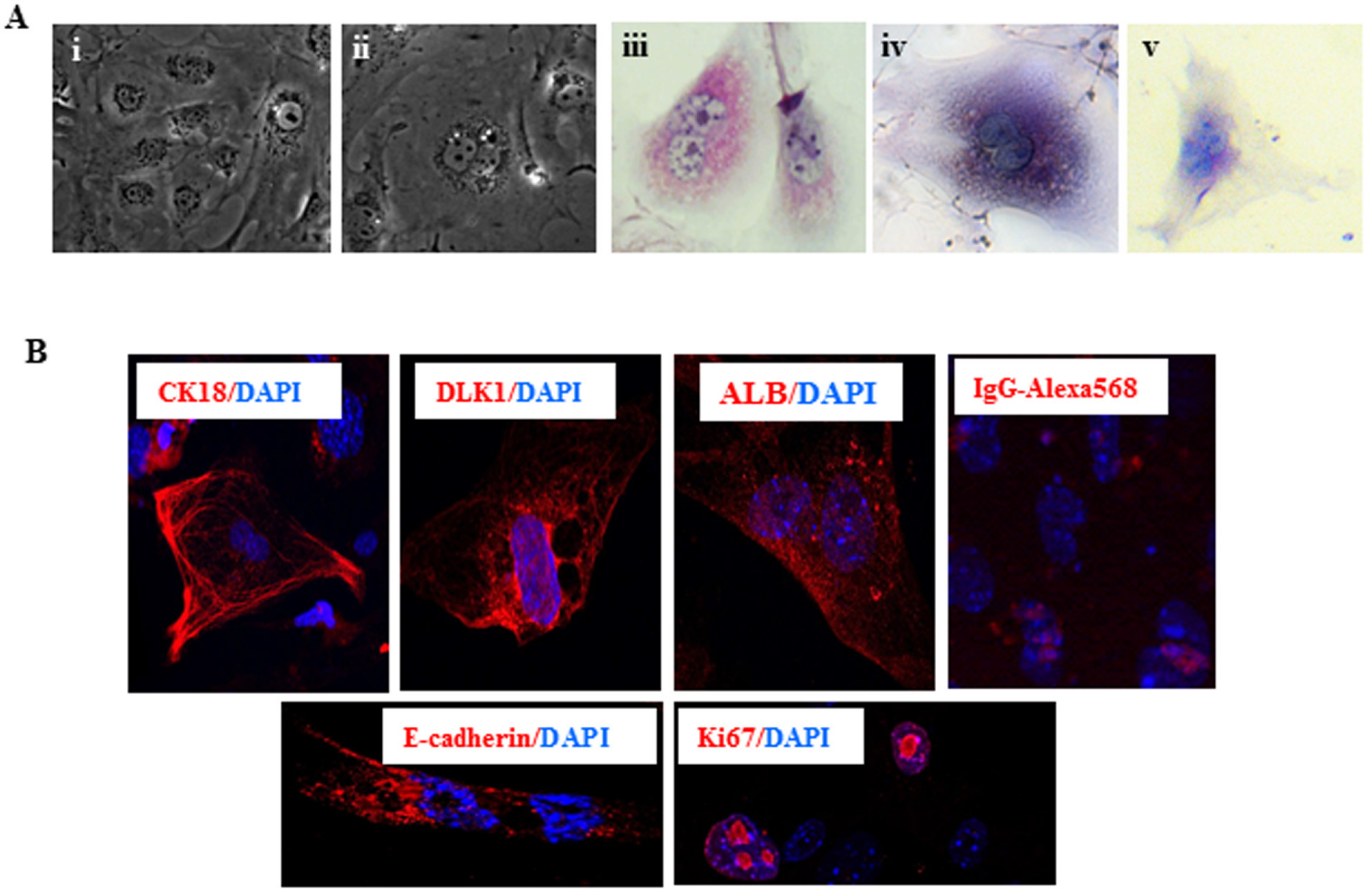
Characterisation of Liv2-sorted GPSC-derived hepatocytes. **A.** Liv2-sorted cells show the typical hepatocyte morphology: binuclear cells with prominent cytoplasm (i, ii) and are positive for glycogen deposits as revealed by PAS staining (iii). PAS staining of non-sorted GPSC-derived hepatocytes are shown as positive control (iv) while mouse embryonic fibroblasts were used as negative control (v) **B.** Immunostaining of Liv2-sorted cells showed positivity for hepatocyte markers CK18, Dlk1, albumin and E-cadherin. These cells were also positive for Ki67, a proliferation marker. Cells were stained with secondary antibody (IgG-Alexa568) only for negative control. Representative images are shown.

Liv2-sorted GPSC-derived hepatocytes were morphologically similar to mouse hepatocytes and were mostly binuclear with prominent cytoplasm (Fig 3Ai and 3Aii). PAS staining showed glycogen deposits in Liv2-sorted GPSC-derived hepatocytes comparable to those of non-sorted GPSC-derived hepatocytes (positive control) (Fig 3Aiii and 3Aiv, respectively). Mouse embryonic fibroblasts, used as negative control, had no glycogen depostis (Fig 3Av). Immunofluorescence analysis showed that these cells were positive for CK18, Dlk1, Albumin and e-cadherin as well as for the proliferation marker Ki67 (Fig 3B). These results indicate that the Liv2-sorted GPSC-derived cells indeed differentiated into hepatocytes as previously reported for unsorted GPSCs.

**Fig 4:**
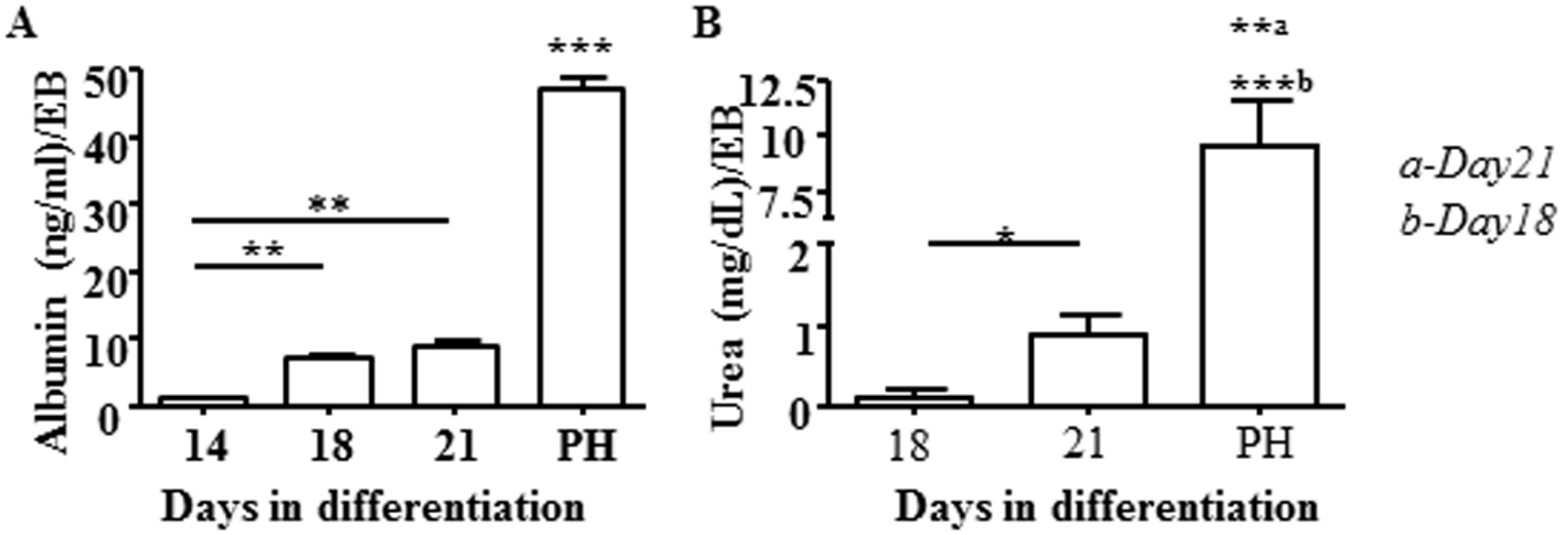
Assessment of functionality *in vitro* of Liv2-sorted GPSC-derived hepatocytes. Functionality was assessed by albumin secretion (A) and urea synthesis (B). Liv2-sorted GPSC- derived hepatocytes were analysed for the presence of albumin (A) and for the metabolism of ammonium chloride into urea (B) at 21 days of induced differentiation. Postnatal hepatocytes (PH) were used as positive control. Data shown are representative of 3 independent experiments performed in triplicate.

In order to assess the functionality of these cells, we collected culture supernatant of Liv2-sorted GPSC-derived hepatocytes grown on collagen gel and tested the presence of albumin by ELISA in comparison to cultured primary hepatocytes (PH). A statistically significant increase in albumin secretion was observed on Day 18 of differentiation and this trend was maintained until Day 21, the last time point analysed (Fig 4A). We also assessed for the production of urea by these cells and by primary hepatocytes. There was a significant increase in urea synthesis from Day18 to Day 21 in Liv2-sorted GPSC-derived hepatocytes, indicating that these cells were functional (Fig 4B). Thus, these GPSC-derived hepatocytes reproduce key features of the primary mouse hepatocytes, albeit exhibiting a not fully mature phenotype.

### *In vivo* engraftment of Liv2-sorted GPSC-derived hepatocytes in mouse livers

We next explored the engraftment and long term residence of the Liv2-sorted GPSC-derived hepatocytes in mice livers. Hfe-null mice, which are a knockout mouse model for hereditary hemochromatosis and which accumulate iron in the hepatocytes, were used for this purpose [19]. Since GPSCs, despite their low level of major histocompatibility complex (MHC) class I molecules on the surface, can become the target for cytotoxic T lymphocytes in case of allogenic transplantation[20], we used GPSCs-derived cells and recipient mice of the same H2^b^ haplotype, thus minimising the risk of reject. Partial hepatectomy was performed on female Hfe-null mice, in which the endogenous hepatocyte proliferation was blocked by treatment with the alkaloid monocrotaline, and 200 000 differentiated cells were injected in one of the remaining liver lobes [21]. Mice were sacrificed at 5 days and 1 month after cell injection. First, livers collected from mice 1 month after injection were inspected for any visible teratomas. We did not observe any teratoma upon injection of Liv2-sorted GPSCs (not shown) whereas, when undifferentiated GPSCs were used, visible teratomas composed of tissues derived from the 3 germ layers (S3 Fig, *) formed, and immunohistochemistry analysis showed positivity for Liv2 (S3 Fig, arrowheads).

**Fig 5:**
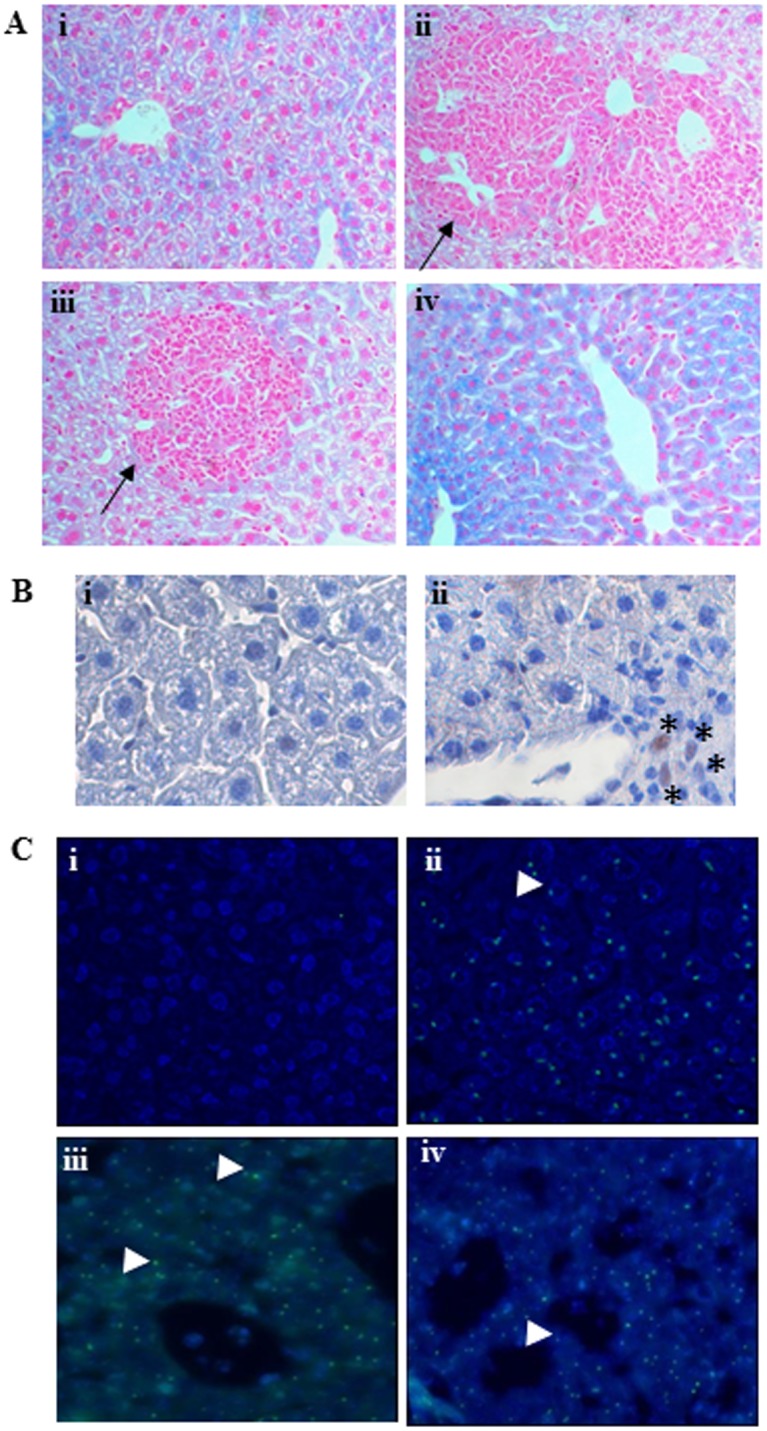
Engraftment of Liv2-sorted GPSC-derived hepatocytes in mouse liver 5 days after injection. A: Engraftment of GPSC-derived hepatocytes in Hfe-null mice livers 5 days post injection. Paraffin-embedded liver sections from control mice were stained with Perls which revealed iron deposits (cytoplasmic blue staining) in endogenous hepatocytes (i) (Magnification:10X). Several areas of regeneration (negative for Perls staining) were evident in the livers of Hfe-null mice which underwent cell tranplantation (ii, iii, arrows). Engraftment was regional and heterogenous; some areas of the injected lobes did not show visible signs of regeneration (iv). B. Anti- PCNA staining of liver sections from Liv2-sorted cells-injected mice to check for cell proliferation. The endogenous hepatocytes (i) do not proliferate while proliferation of cells (ii, *) in the Perls’-negative, regenerating areas was observed. C. Fluorescent in situ hybridization (FISH) was performed to analyse for the presence of Y chromosome (green dots). The Perls’-negative cells were positive for Y-chromosome (iii,iv, arrowheads), indicating the provenience of these cells compared to untreated female livers (i). Male livers were used as positive control for FISH(ii). Representative images are shown (n = 6).

Analysis of liver sections at 5 days post-injection showed that there were regenerating areas around central veins and venules (Fig 5Aii and 5Aiii) compared to PBS-injected controls (Fig 5Ai). Perls staining of liver sections revealed that while Hfe-null mice hepatocytes showed prominent iron deposits, the regenerating areas were negative for iron accumulation in the cytoplasm at 5 days (Fig 5Aii and 5Aiii). Engraftment was regional and heterogenous in the Liv2-sorted cells-injected liver lobe (Fig 5Aii, 5Aiii and 5Aiv). Immunohistochemistry on liver sections with an anti-PCNA antibody, used to check for cell proliferation, showed PCNA-positive cells in the regenerating, Perls’-negative (Fig 5Bii, *) regions, while endogenous hepatocytes ((Fig 5Bi) were PCNA-negative indicating that they did not proliferate. In order to track the Liv2-sorted, male GPSC-derived, hepatocytes *in vivo*, Y chromosome FISH was performed on female mice liver sections. At 5 days post-injection, Y chromosome-positive and Perls-negative cells could be detected in the regenerating areas in the Liv2-sorted cells-injected female mice livers (Fig 5Ciii and 5Civ, arrowheads) compared to the PBS-injected female mice livers (Fig 5Ci). A male mouse liver was used as positive control (Fig 5Cii).

**Fig 6:**
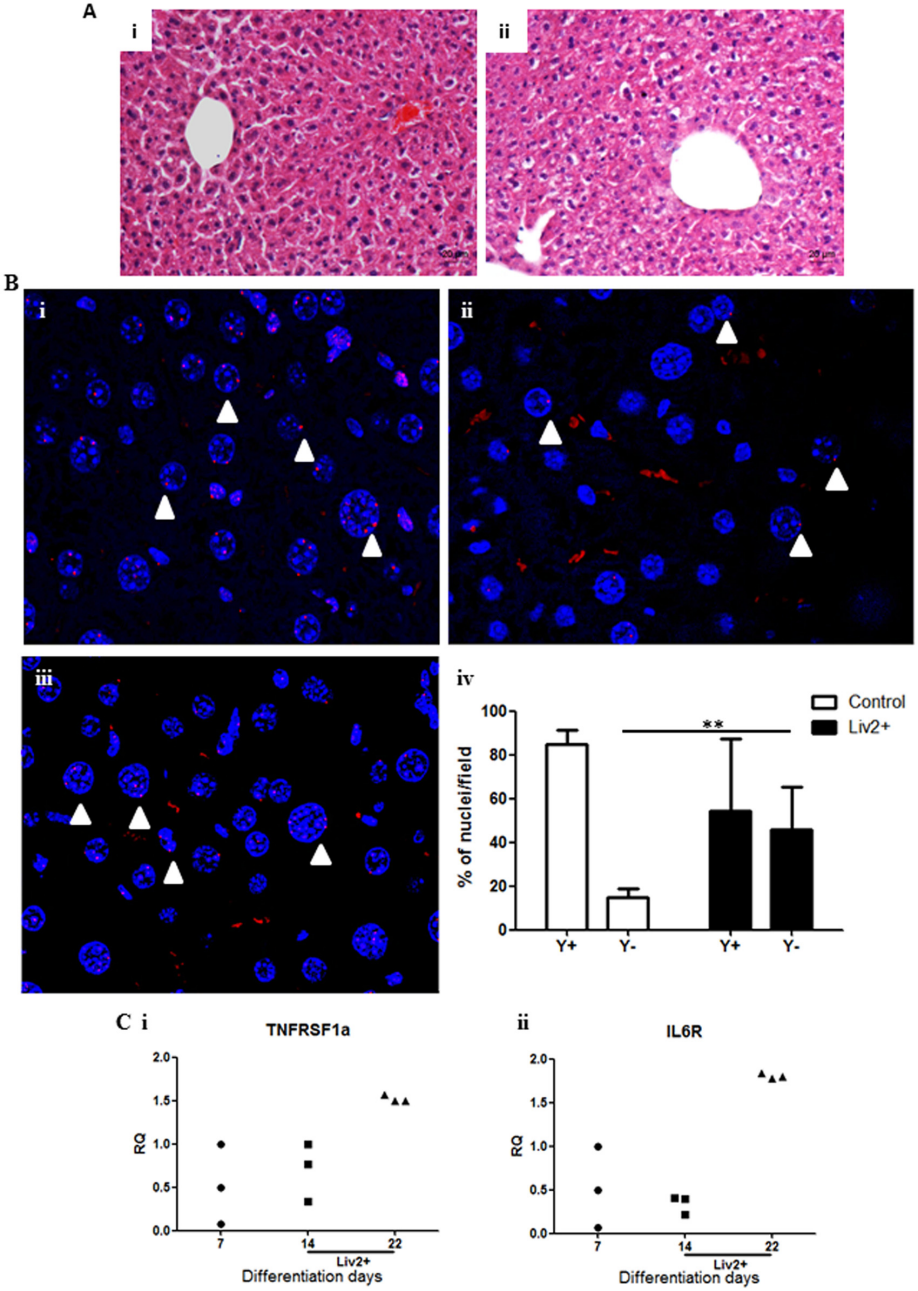
Engraftment of Liv2-sorted GPSC-derived hepatocytes in mouse liver 1 month after injection. A. A representative Hematoxylin/Eosin staining of the liver of Liv2-sorted GPSC-derived hepatocytes injected mice is shown (ii) and compared to that of non-treated mice (i). (Magnification:10X). B. FISH analysis showed the presence of Y chromosomes (red dots, arrowheads) in the liver of a control male mouse (i) and in the liver of female mice injected with Liv2-sorted cells (ii, iii). Representative images are shown (n = 4). The percentage of Y chromosome-positive cells and of Y chromosome-negative cells per field (138x104μm^2^) is shown (iv) for the Liv2-sorted cells-injected female livers at 1 month post-transplant compared to that of male mice livers (n = 6). C. qRT-PCR shows increased expression of TNFRSF1A (i) and IL6R (ii) in Liv2-sorted cells at Day 22 of differentiation compared to Day7 EBs and Day14 Liv2-sorted cells (n = 3). The relative quantity (RQ) with respect to gene expression in EBs at Day 7 of differentiation is shown and values have been normalized to 18S expression.

At 1 month post-injection (the last time point analysed), we did not find any inflammatory foci or signs of necrosis in the liver of mice injected with Liv2-sorted GPSC-derived hepatocytes (Fig 6A). However, the hepatocytes in the injected lobes were no longer arranged in plates or chords and there were varying degrees of glycogen storage which appear as unstained areas in the hematoxylin/eosin stained sections (Fig 6Aii) compared to the PBS-injected livers (Fig 6Ai). This was probably the consequence of the cell injection and the complexity of the regenerative process [22, 23]. FISH analysis revealed the presence of a substantial number of Y chromosome-positive nuclei in the liver of the female Hfe-null mice showing long term engraftment of these cells *in vivo* (Fig 6Bii, 6Biii and S4 Fig). A male mouse liver was used as positive control (Fig 6Bi and S4 Fig). Y chromosome FISH at 1-month post-injection confirmed that engraftment was regional and heterogeneous as demonstrated by the comparison between liver sections of Liv2-sorted cells-injected female mice, showing scattered areas containing Y-positive nuclei, and sections of a male mouse control liver showing positivity in all cells. In the regenerated areas of female livers, there were 54.34% ± 33.06 of Y chromosome-positive and 45.66% ± 19.84 of Y-chromosome–negative cells per field (138x104μm^2^) while in the male liver, there were 84.98% ± 7.31 of Y chromosome-positive and 15.02% ± 4.26 of Y chromosome-negative cells per field (138x104μm^2^) (Fig 6Biv). These data show that the Liv2-sorted GPSC-derived hepatocytes participated in the regenerative process in the female mice livers.

As during hepatectomy, usually, several cytokines are released that may participate in homing of cells to the damaged liver, like for e.g., tumor necrosis factor (TNF) and interleukin 6 (IL6), the Liv2-sorted GPSC-derived hepatocytes were analysed for the expression of TNF receptor (TNFR) and IL6 receptor (IL6R). In particular, the expression of tumor necrosis factor receptor superfamily, member 1a (TNFRSF1A) and interleukin 6 receptor, alpha (IL6RA) was significantly upregulated in the Liv2-sorted hepatocytes compared to Days 7 and 14 non-sorted ones (Fig 6Ci and 6Cii, respectively), indicating a mechanism through which these cells can home to the damaged liver.

## Discussion

Research on pluripotent stem cells derived from the testis is still in its early days, but it may offer an alternative to ES or iPS cell-based therapies. In the present study, we show that Liv2-sorted GPSC-derived hepatocytes are functional *in vitro*, and express hepatocyte-specific genes, secrete albumin and synthesize urea. Moreover, these cells can home and engraft in mouse livers and participate in liver regeneration after partial hepatectomy.

There are several ways of inducing differentiation of pluripotent stem cells *in vitro* to make these adopt a specific cell fate, like the formation of EBs followed by treatment with a cocktail of growth factors or through direct differentiation [24–26]. In the present work, to induce the GPSCs to differentiate into hepatocytes, we generated EBs by culturing the cells in suspension, followed by induction of definitive endoderm from 2 day-old EBs, hepatic specification and hepatocyte maturation. EBs are three-dimensional aggregates comprised of the three germ layers and recapitulate the early steps of embryonic development. EB differentiation is a routine way of generating specific lineages from pluripotent stem cells *in vitro*[27]. One of the major concerns about using EBs for differentiation, however, is the heterogenous population of cells that is generated and the presence of remaining undifferentiated cells [28]. In order to avoid teratoma formation *in vivo*, we sorted the hepatocyte precursors by using Liv2 as a fetal liver cell surface marker which can be used to isolate immature hepatocytes [17, 29]. The advantage of using immature compared to fully mature hepatocytes for *in vivo* colonisation experiments is that the former have a higher proliferative capacity with respect to the latter [30]. The Liv2-sorted GPSC-derived hepatocytes expressed hepatocyte-specific proteins at Day 21 of differentiation and were functional in that they were able to secrete albumin in culture supernatant as well as produce urea. These cells also expressed glucose-6-phosphatase, catalytic subunit gene as shown in S5 Fig, reflecting their differentiation status. After Liv2-sorting, we had an enrichment in the number of hepatocytes compared to unsorted GPSCs (82.62% *versus* 63.1%, respectively, at Day 20 of differentiation). Most importantly, the sorted cells were completely Oct4-negative compared to the previously described non-sorted GPSCs [16]

In order to study engraftment of these cells *in vivo*, we used the well-characterised Hfe-null mice[31]. Hfe is a membrane protein that modulates iron absorption by regulating the interaction of transferrin receptor with its substrate, transferrin. Usually the hepatocytes in an adult liver regenerate only sparsely under physiological conditions. However, after partial hepatectomy, the hepatocytes in the remaining lobes are capable of undergoing cell division, with a peak of DNA synthesis occurring at 36 hours after surgery in the mouse, to compensate for the missing mass. We therefore blocked the endogenous hepatocyte proliferation in our mice so that the liver could only repopulate using the transplanted Liv2-sorted cells. There are several ways of injecting cells in the mouse to investigate homing and engraftment into the liver: intravenous, intrasplenic and intraparenchymal. We chose to directly deliver Liv2-sorted cells into the liver parenchyma of Hfe-null mice in order to study engraftment. Regenerating areas were found in Hfe-null mice livers as early as 5 days after injection of GPSC-derived hepatocytes. Our FISH results showed that the male Liv2-sorted GPSC-derived hepatocytes were still present in the female mice livers after partial hepatectomy at one month (the last time point analysed) post-injection and participated to the regenerative process. The engraftment was regional and heterogenous in the injected liver lobes.

Partial hepatectomy is a safe and effective model for investigating the engraftment of hepatocytes[22]. The liver regeneration induced after partial hepatectomy may help in the homing of injected cells through release of TNFα and IL6 for instance [32]. Interestingly, our large scale gene expression profiling on GPSCs during hepatocyte differentiation *in vitro*, revealed that the expression of TNFRSF1A and IL6RA, receptors for TNFα and IL6, respectively, increased in the differentiated compared to undifferentiated cells [16]. A qRT-PCR analysis confirmed this trend also in the Liv2-sorted GPSC-derived hepatocytes at Day 22 of differentiation. The increase in expression of these receptors may promote homing and engraftment of these cells in the hepatectomised liver.

GPSCs can be differentiated into different cell types and are capable of engrafting in several mouse tissues as demonstrated by several studies. Baba *et al*. showed that Flk1+ GPSC-derived cells transplanted into the heart of ischemic mice improved cardiac function and these cells could be found *in vivo* 4 weeks after treatment[33]. There is also report of GPSC-derived hematopoietic cells homing to bone marrow cavity following intra-bone marrow injection in immune-compromised mice[34]. Moreover, we recently described the engraftment of GPSC-derived renal tubular cells in mice after renal damage [15]. To our knowledge, this is the first study demonstrating the engraftment of GPSC-derived hepatocytes in the liver of mice. Further studies are needed to investigate whether these GPSC-derived cells remain in the liver for a longer time and whether these cells can rescue mice from severe liver diseases that result in lethality when left untreated [15].

## Conclusion

GPSCs are a promising source of hepatocytes that can be easily obtained for cell therapy. Especially, in pediatric patients awaiting transplantation, GPSC-derived hepatocytes may serve as a “bridging” therapy. Moreover, GPSC-derived hepatocytes may serve as a platform for gene delivery and can partially substitute the diseased liver as in case of metabolic diseases requiring only 5–10% healthy cells for rescue, as in the case, for instance, of Crigler-Najjar syndrome type I for which only 5% of the functional Ugt1a1 enzyme can benefit the patients as well as animal models affected by this disease[35]. Assuming translation of the mouse GPSC system onto the human cells, GPSCs may be yet another source of injectable hepatocytes for cell therapy of liver diseases.

## Supporting Information

S1 Fig Staining of EBs with anti-Liv2 antibodies. Liv2 positivity is mainly localised in the EB outgrowths (i, arrowhead) and is observed in Days 11 (ii, arrowheads), 13 (iii, arrowheads) and 15 (iv, arrowheads) GPSC-derived EBs.
(TIF)

S2 Fig Liv2 sorted GPSC-derived hepatocytes express Hfe gene. qRT-PCR shows that Hfe gene was expressed as from Day 14 of differentiation in Liv2-sorted cells (n = 3). The relative quantity (RQ) with respect to gene expression in EBs at Day 7 of differentiation is shown and values have been normalized to 18S expression.
(TIF)

S3 Fig Teratoma formation with undifferentiated GPSCs. Undifferentiated GPSCs form teratomas upon injection in the mouse liver parenchyma. Tissues originating from the 3 germ layers (endoderm, mesoderm and ectoderm, *) are found. Immunohistochemistry reveals positivity for Liv2 in the teratoma sections (IHC: Liv2, arrowheads).
(TIF)

S4 Fig FISH analysis of livers at 1 month after injection of Liv2-sorted cells. FISH analysis shows the presence of Y chromosomes (red dots, arrowheads) in the liver of a control male mouse (i) and in the liver of female mice injected with Liv2-sorted cells (ii, iii). Representative images (magnification: 100x) of the Liv2-cells injected livers are shown (ii, iii).
(TIF)

S5 Fig Analysis of glucose-6-phosphatase, catalytic subunit expression in Liv2-sorted cells. qRT-PCR shows that glucose-6-phosphatase, catalytic subunit (G6Pc) was expressed in Liv2-sorted cells at Day 22 of differentiation compared to Day7 EBs and Day14 Liv2-sorted cells (n = 3). The relative quantity (RQ) is shown and values have been normalized to the expression of G6Pc in EBs at Day 7 of differentiation.
(TIF)
