# Supplementary figures and images for: Long Term Liver Engraftment of Functional Hepatocytes Obtained from Germline Cell-Derived Pluripotent Stem Cells
Source: PLoS One. 2015 Aug 31;10(8):e0136762. doi: 10.1371/journal.pone.0136762 (PMC4556379; doi:10.1371/journal.pone.0136762)

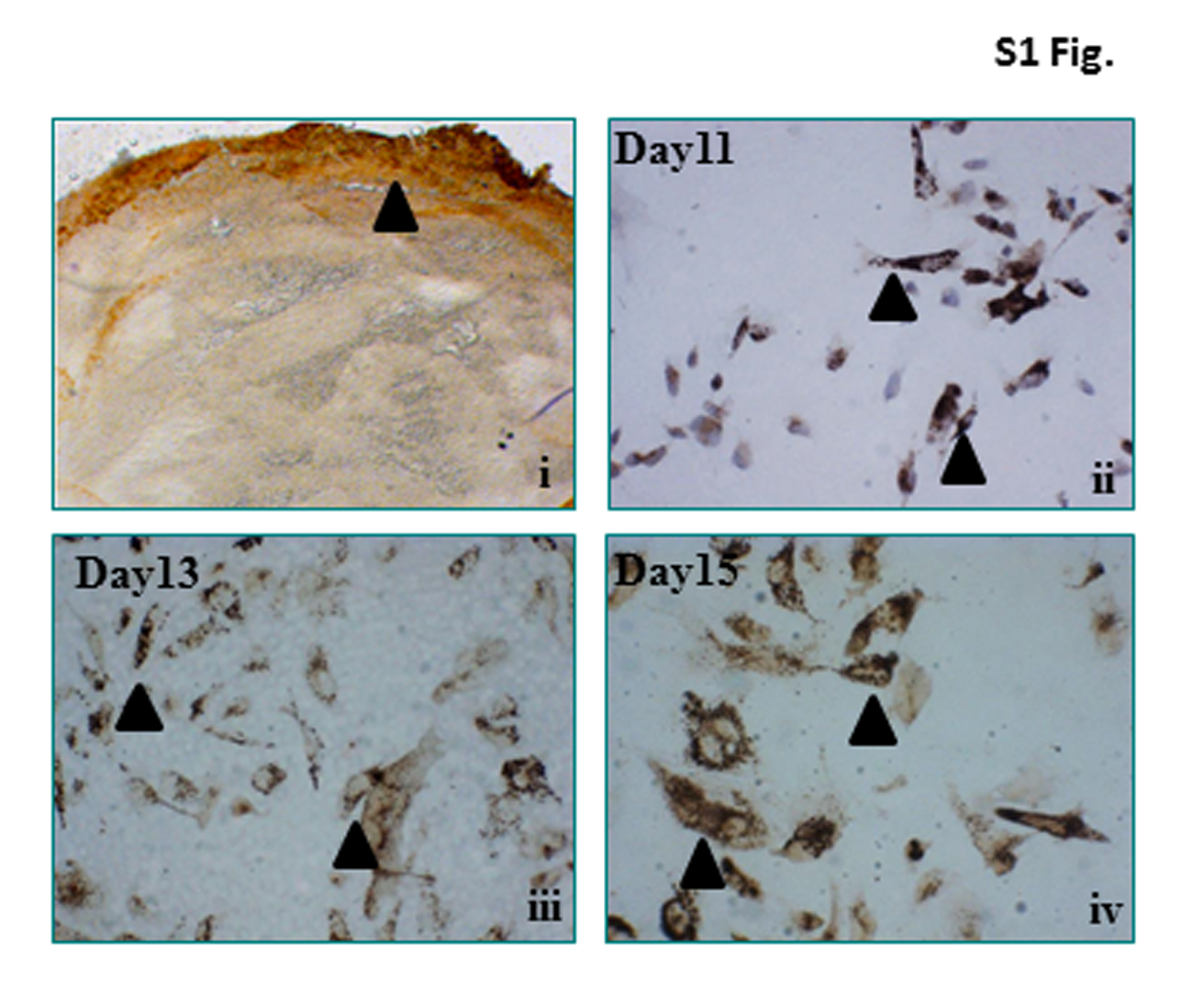

Supplement: S1 Fig — Liv2 positivity is mainly localised in the EB outgrowths (i, arrowhead) and is observed in Days 11 (ii, arrowheads), 13 (iii, arrowheads) and 15 (iv, arrowheads) GPSC-derived EBs. (TIF) [file pone.0136762.s001.tif]

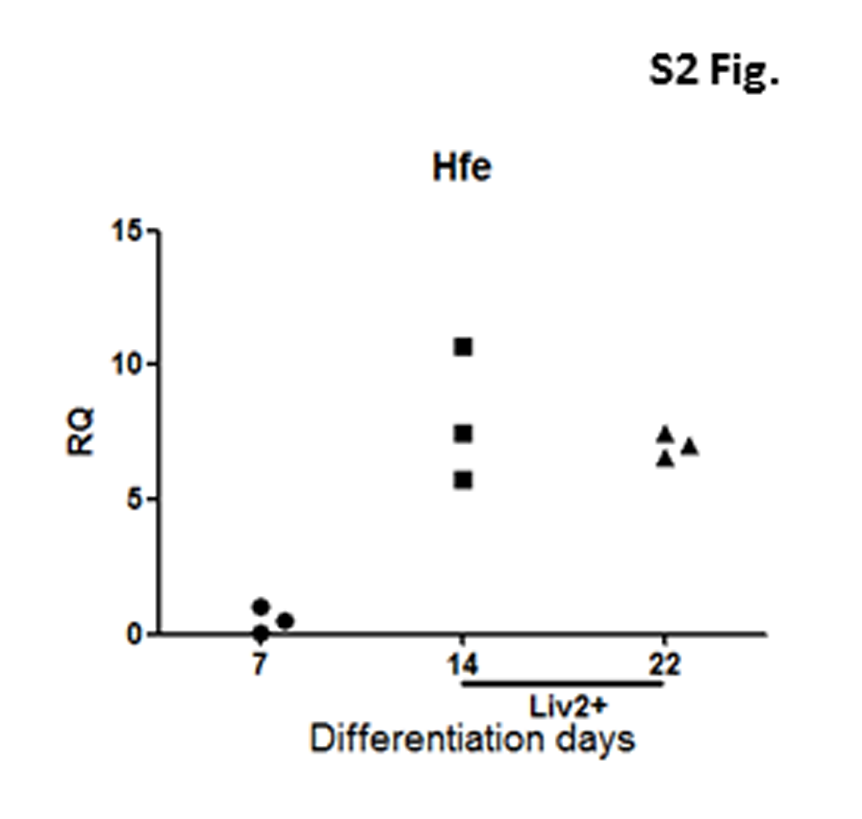

Supplement: S2 Fig — qRT-PCR shows that Hfe gene was expressed as from Day 14 of differentiation in Liv2-sorted cells (n = 3). The relative quantity (RQ) with respect to gene expression in EBs at Day 7 of differentiation is shown and values have been normalized to 18S expression. (TIF) [file pone.0136762.s002.tif]

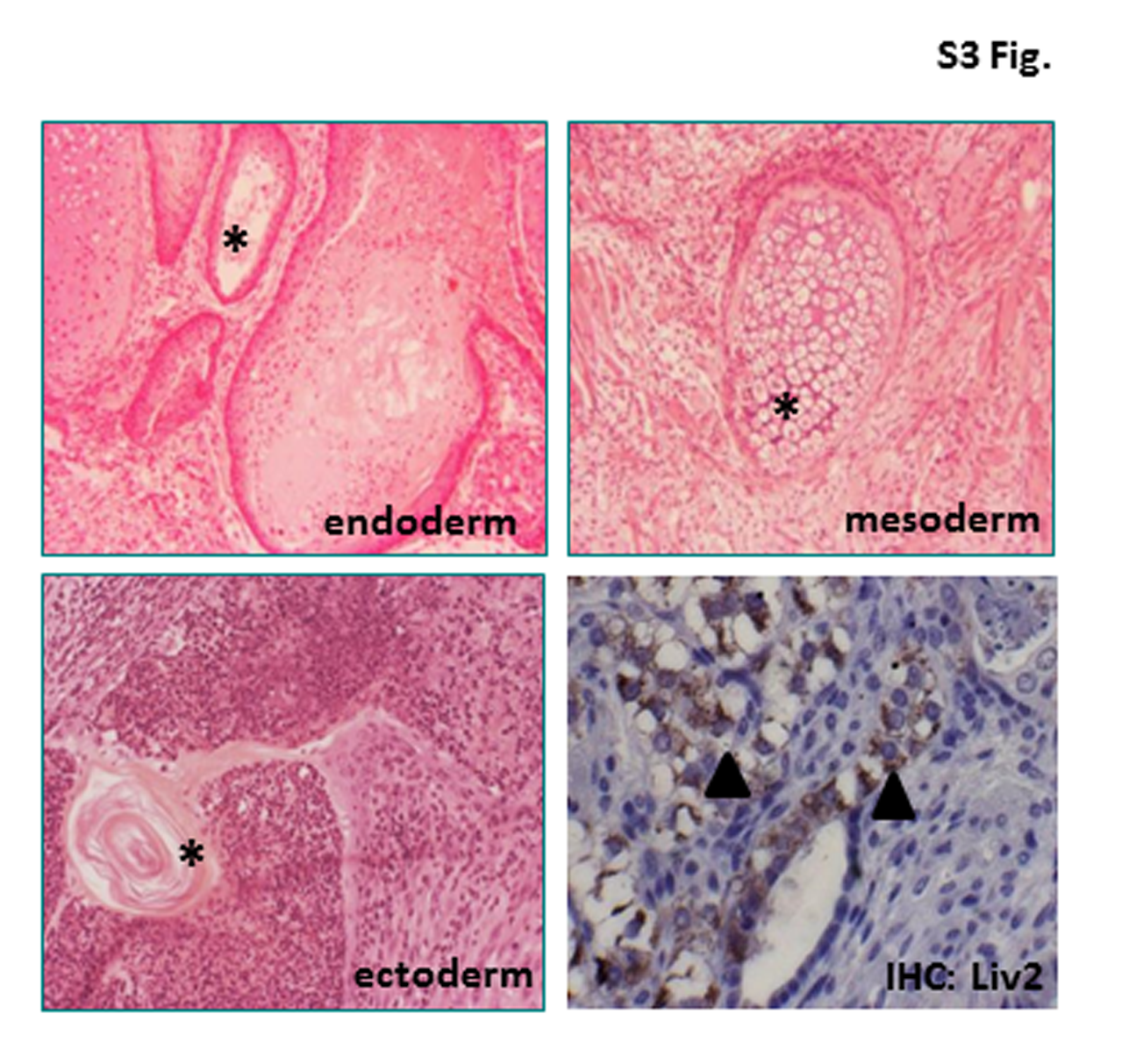

Supplement: S3 Fig — Undifferentiated GPSCs form teratomas upon injection in the mouse liver parenchyma. Tissues originating from the 3 germ layers (endoderm, mesoderm and ectoderm, *) are found. Immunohistochemistry reveals positivity for Liv2 in the teratoma sections (IHC: Liv2, arrowheads). (TIF) [file pone.0136762.s003.tif]

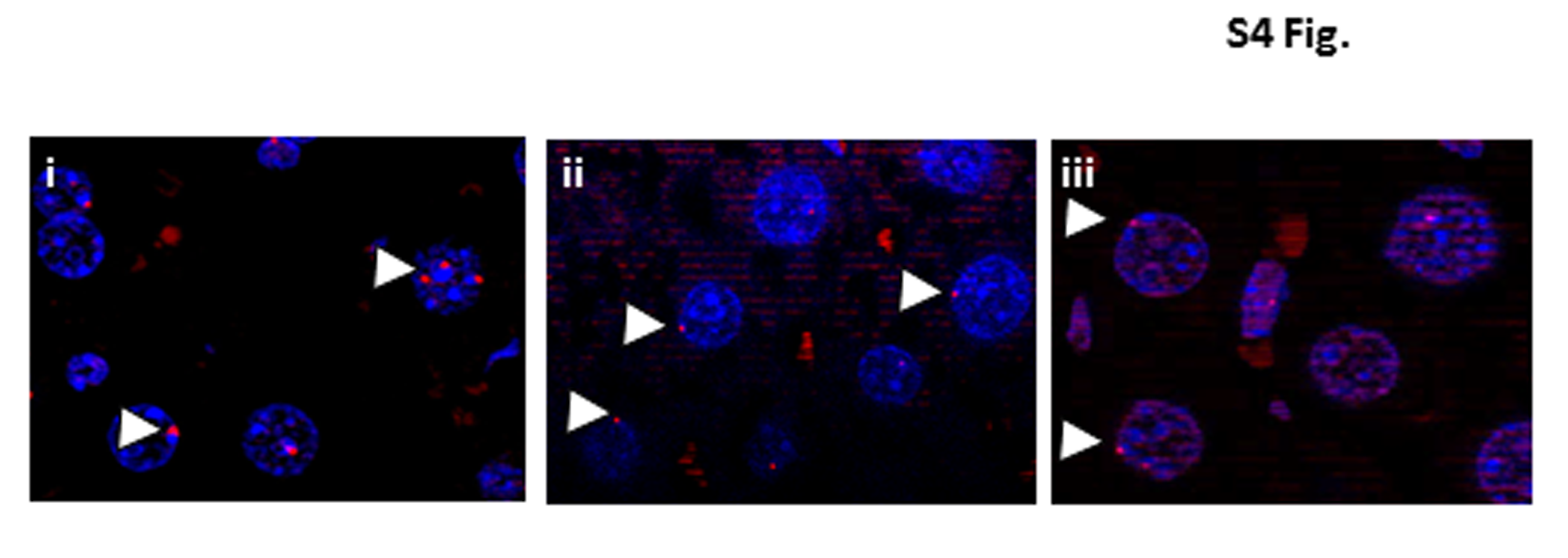

Supplement: S4 Fig — FISH analysis shows the presence of Y chromosomes (red dots, arrowheads) in the liver of a control male mouse (i) and in the liver of female mice injected with Liv2-sorted cells (ii, iii). Representative images (magnification: 100x) of the Liv2-cells injected livers are shown (ii, iii). (TIF) [file pone.0136762.s004.tif]

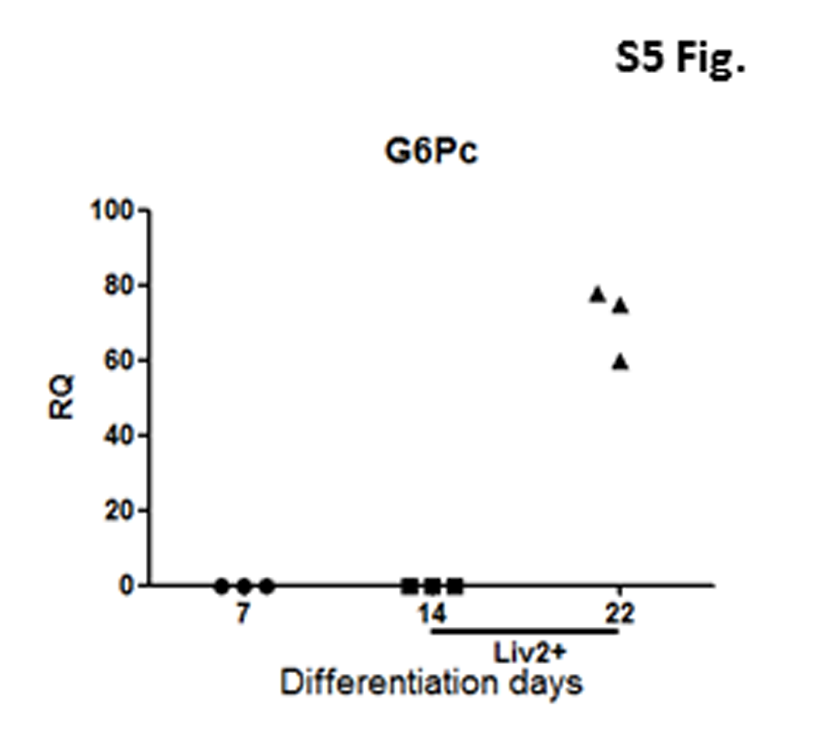

Supplement: S5 Fig — qRT-PCR shows that glucose-6-phosphatase, catalytic subunit (G6Pc) was expressed in Liv2-sorted cells at Day 22 of differentiation compared to Day7 EBs and Day14 Liv2-sorted cells (n = 3). The relative quantity (RQ) is shown and values have been normalized to the expression of G6Pc in EBs at Day 7 of differentiation. (TIF) [file pone.0136762.s005.tif]
